# One step at a time: new insights into double-stranded ribonucleic acid virus assembly

**DOI:** 10.1038/s41392-024-01949-3

**Published:** 2024-09-13

**Authors:** Virgile Rat, Alexander Borodavka, Don C. Lamb

**Affiliations:** 1 Department of Chemistry, LMU München, München Germany, Butenandtstrasse 5-13, 81377 Munich, Germany; 2 Department of Chemical Engineering and Biotechnology, University of Cambridge, Philippa Fawcett Drive, CB3 0AS Cambridge, UK

**Keywords:** Biophysics, Structural biology

In a recent paper published in *Cell*, Xia et al.^1^ carried out 3D reconstructions of Blue Tongue Virus (BTV) particles and their assembly intermediates using a combination of cellular cryo-electron tomography (Cryo-ET) and single-particle cryo-electron microscopy (Cryo-EM). They were able to identify 11 structures, allowing them to refine our understanding of how double-stranded ribonucleic acid (dsRNA) viruses replicate in cells.

RNA viruses infect all kingdoms of life, shaping their evolution and causing devastating pandemics. Amongst these pathogens, a fascinating and diverse group of RNA viruses incorporate a single copy of multiple non-identical dsRNA molecules referred to as gene segments. Some notable examples of these important infectious agents include rotaviruses, avian and mammalian reoviruses, and BTV. Importantly, BTV has been studied extensively as it can have devastating consequences for livestock and is an excellent model system for dissecting the replication mechanisms of all segmented dsRNA viruses. The exact assembly mechanisms dsRNA viruses have remained an unsolved mystery for several decades. Two models of morphogenesis of segmented dsRNA viruses have been proposed based on extensive biochemical and structural studies of these pathogens.^2^ The concerted, or co-assembly model, proposes that the viral genome (gRNA) and the polymerase complexes are encapsidated during the co-assembly of viral capsid proteins. In contrast, the core-filling model postulates that the inner capsid of multilayered dsRNA viruses assembles prior to the gRNA being threaded into the capsid. Unveiling virus assembly steps remains an extremely difficult task often due to the low stability and transient nature of assembly intermediates in the background of many kinetically trapped misassembled products. This becomes even more challenging when studying virus assembly mechanisms in situ.

**Fig. 1:**
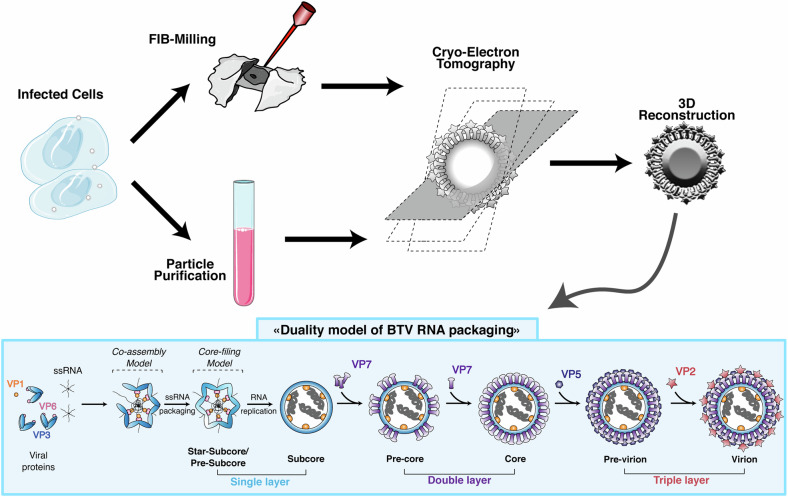
BTV duality model based on a combination of observations in situ and from purified particles using cryo-electron tomography. Infected cells were either grown on EM-grids to be FIB-milled or used for viral particle purification prior to cryo-electron tomography. The 3D reconstruction of the tomograms led to the characterization of assembly intermediates, helping the authors to propose a duality model where RNAs interact with each other and bind to the viral proteins VP3&VP6 to promote the capsid formation. The star-subcore and the pre-subcore undergo expansion following the remainder of each ssRNA threading into the capsid, leading to the displacement of VP6 by VP1, followed by the VP7 coating of the particle. (The cell and test tube images were taken from SMART (Servier Medical ART) resource pictures and used under the open access creative commons license CC BY 4.0 https://creativecommons.org/licenses/by/4.0/)

The group of Zhou at UCLA rose to this challenge. Initially, they investigated the structure of BTV during assembly using cellular cryo-ET and refined the structures using purified particles. The BTV virion is composed of three layers of viral proteins. In particular, the authors highlighted the single-layered star-shaped particle with two conformations formed by the capsid protein VP3 with a diameter of around 55 nm, termed the star-subcore and pre-subcore, and an expanded version of the particle, termed the subcore (Fig. 1). Upon assembly, a second, double-layered particle, with a diameter of 68 nm, was determined, which they identified as a core, and finally, the triple-layered 72-nm-diameter particle was observed. All states were found inside the cytoplasmic viral inclusion bodies (VIBs), or viral factories, further supporting the notion that VIBs serve as sites of virus assembly in cells. To increase the resolution of the individual structures, the authors performed single-particle cryo-EM reconstruction of intermediates purified from infected cells via centrifugation through a sucrose cushion. The authors note that the fraction of subcore particles was less than 1% in comparison to the previous and following intermediate states in the isolates, indicating the transient nature of subcore particles. Less than 20% of all particles analyzed were devoid of RNA, which the authors attribute to the important role of gRNA in virion assembly. The analysis revealed that the electron density corresponding to RNA in the subcore is similar to that observed in the core and virion itself, suggesting that the genome has already been replicated from single-stranded to dsRNA in the subcore. Remarkably, the star-subcore of BTV as well as the pre-subcore are reminiscent of the star-shaped particles also recently reported for reoviruses^3^ and rotaviruses.^4^

This suggests that the RNA encapsidation likely occurs during the pre-subcore formation. Therefore, the authors focused on these intermediates and identified the presence of the RNA helicase VP6 at the position where the RNA-dependent RNA Polymerase VP1 is observed in other states. Furthermore, a density corresponding to the ssRNA was also seen in this state, both inside the capsid and in an 18 Å-wide tunnel at the fivefold axis of symmetry created by the VP3-VP6 pentamers. Importantly, the authors observed that no VP1 was encapsidated in the virions lacking gRNA, suggesting that the gRNA-VP1 interactions are essential for their packaging in BTV.

By combining cellular cryo-ET and single-particle cryo-EM of isolated states, the authors characterized eleven distinct assembly states of the BTV particles. It is possible that not all the structures represent productive assembly intermediates of the infection cycle, and additional assembly states could have been missed due to their short-lived transient nature. Particularly, the most enigmatic intermediates formed in the earliest stages of viral morphogenesis that involve interactions with the ten distinct ssRNAs comprising the pre-genome. Capturing these early states requires methods that can follow the dynamics of RNA packaging in live cells with high temporal resolution such as single-particle tracking or single-molecule FRET.^5^ These methods in combination with cryo-EM imaging of viral replication promise to reveal insights into the spatio-temporal dynamics of viral assembly. Such data will be invaluable for structure-based hypotheses generation that can be both tested in silico and, importantly, in vitro, thanks to progress in the development of fully tractable reverse genetics systems for these viruses. Nevertheless, these exciting results have provided new insights into the assembly process of BTV, revealing several interesting parallels with genome packaging mechanisms in other viruses. This includes the large family of *Reoviridae* viruses and dsRNA phages of the *Cystoviridae* family, which insert their ssRNAs into preformed star-shaped particles before they undergo expansion due to RNA synthesis.
